# A DNA origami-based nanoscale molecular transport railway

**DOI:** 10.1038/s42003-021-02579-z

**Published:** 2021-09-09

**Authors:** Theam Soon Lim, Karli Montague-Cardoso

**Affiliations:** 1 Institute for Research in Molecular Medicine, Universiti Sains Malaysia, Gelugor, Malaysia; 2 Communications Biology, http://www.nature.com/commsbio

## Abstract

Origami, the Japanese art of paper folding, has taken on new meaning for the fields of chemistry and biology. DNA origami describes the folding of DNA strands to form nanoscale structures. The ability to design and form complex structures at a nanoscale level has fuelled new ambitions of nanostructure applications in life science. These predefined shapes become base structures for the development of a higher and complex functional structure. In a recent paper, Stömmer et al., demonstrated the ability to design a macromolecular level transportation network that allows the movement of molecules at sub-molecular levels using DNA. A multi-layer DNA origami was used to build micrometer-long hollow tunnels akin to railway tunnels. An accompanying DNA piston travelled through the tunnels with constant motion. The system also accommodated the application of electric fields to fuel the motion of the pistons along the filaments simulating a nanoscale electric railway system. This could revolutionize the way molecular drug delivery systems can be perceived in the future.

**Figure:**
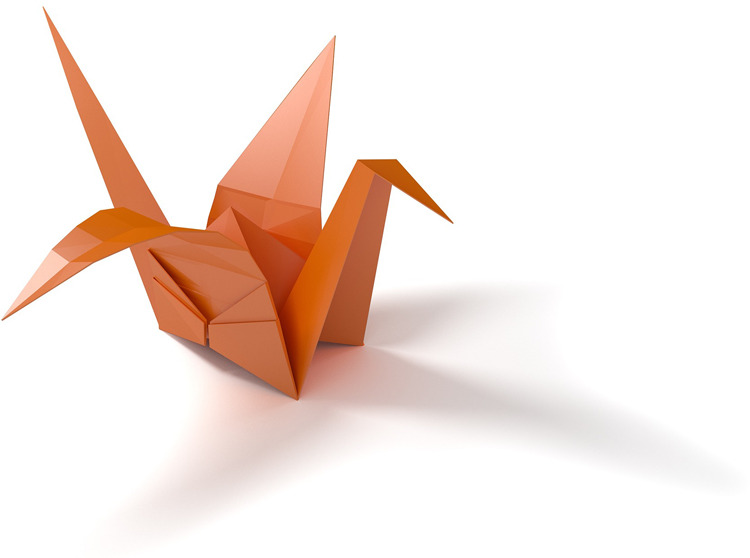
Pixabay

The double helix DNA structure allows DNA origami designers to develop complex constructs using strands of DNA. The developments in DNA origami technology have seen DNA nanostructures being used initially for the construction of 1-dimensional structures to now more complex, higher order structures. These nanostructures have been applied to many applications including studying enzyme-substrate interactions, molecular motor actions, drug delivery, fluorescence and nanorobotics.

Recently, Stömmer et al. described the design of a hollow filament structure using multi-layer DNA origami to propel a molecular piston^1^. The authors applied a DNA-based, programmable self-assembly system that replicates the intrinsic properties of molecular transporters in nature. This system includes multiple-micrometer-long travel ranges with μm/s displacement speeds and is an evolution of other known artificial molecular devices and machines like pivots, hinges, crank sliders, pliers and rotors. The authors observed the piston motions within the filaments using single-molecule fluorescence microscopy and performed a dynamics simulation to determine the free energy surface that fuels the movement of the pistons. They reported that the diffusive and displacement ranges of this new system were within the ranges of natural molecular motors but with five orders of magnitude of improvement over existing artificial random walker designs. The system also showed adaptability in different environments and stimuli, and the work also showcased the possibility of applying electric fields to fuel the linear motion of the DNA piston over a long range along molecular tracks at high speeds.

The published system is a marked improvement over traditional propulsion mechanisms used in DNA origami structures. The authors were able to stretch the boundaries of distance and speed with this new design thus broadening the realm of possible applications of this technology.
